# Validation of Gene Expression-Based Predictive Biomarkers for Response to Neoadjuvant Chemoradiotherapy in Locally Advanced Rectal Cancer

**DOI:** 10.3390/cancers13184642

**Published:** 2021-09-16

**Authors:** Tomoyuki Momma, Hirokazu Okayama, Yasuyuki Kanke, Satoshi Fukai, Hisashi Onozawa, Shotaro Fujita, Wataru Sakamoto, Motonobu Saito, Shinji Ohki, Koji Kono

**Affiliations:** 1 Department of Gastrointestinal Tract Surgery, Fukushima Medical University School of Medicine, 1 Hikarigaoka, Fukushima 960-1295, Japan; tmomma@fmu.ac.jp (T.M.); kanke33@fmu.ac.jp (Y.K.); sfukai@fmu.ac.jp (S.F.); hisa444@fmu.ac.jp (H.O.); newyork@fmu.ac.jp (S.F.); ws1024@fmu.ac.jp (W.S.); moto@fmu.ac.jp (M.S.); ohki@fmu.ac.jp (S.O.); kojikono@fmu.ac.jp (K.K.); 2 Hospital Director, Shirakawa Kosei General Hospital, 2-1 Kamiyajiro, Shirakawa, Fukushima 961-0005, Japan

**Keywords:** locally advanced rectal cancer, neoadjuvant chemoradiotherapy, predictive biomarker, pre-treatment biopsy, gene expression signature, microarray, meta-analysis

## Abstract

**Simple Summary:**

There is a clinical need for predictive biomarkers that can identify patients with rectal cancer who do not respond to preoperative neoadjuvant chemoradiotherapy. In this study, we assembled multiple independent microarray datasets of biopsy specimens obtained from patients with rectal cancer before neoadjuvant treatment, including 237 non-responders and 152 responders. These datasets were utilized as the discovery cohorts or the validation cohorts, to develop and validate gene expression signatures predictive of treatment response. Using an in silico meta-analysis approach, here we tested not only our 4-gene signature built in this study but also nine different single-gene and multi-gene predictive signatures that were previously reported in the literature. Nevertheless, in the validation cohorts, none of the tested signatures were consistently differentially expressed between tumor specimens from non-responders and responders, and the meta-analyses revealed that those signatures had limited predictive values in clinical practice.

**Abstract:**

Background: Neoadjuvant chemoradiotherapy (nCRT) followed by surgery is widely used for patients with locally advanced rectal cancer. However, response to nCRT varies substantially among patients, highlighting the need for predictive biomarkers that can distinguish non-responsive from responsive patients before nCRT. This study aimed to build novel multi-gene assays for predicting nCRT response, and to validate our signature and previously-reported signatures in multiple independent cohorts. Methods: Three microarray datasets of pre-therapeutic biopsies containing a total of 61 non-responders and 53 responders were used as the discovery cohorts to screen for genes that were consistently associated with nCRT response. The predictive values of signatures were tested in a meta-analysis using six independent datasets as the validation cohorts, consisted of a total of 176 non-responders and 99 responders. Results: We identified four genes, including *BRCA1*, *GPR110*, *TNIK,* and *WDR4* in the discovery cohorts. Although our 4-gene signature and nine published signatures were evaluated, they were unable to predict nCRT response in the validation cohorts. Conclusions: Although this is one of the largest studies addressing the validity of gene expression-based classifiers using pre-treatment biopsies from patients with rectal cancer, our findings do not support their clinically meaningful values to be predictive of nCRT response.

## 1. Introduction

Colorectal cancer remains one of the leading causes of cancer death worldwide, with rectal cancer accounting for one-third of these cases [1]. During the past decade, advances in treatment strategies with the use of standardized surgical technique, combined with preoperative (neoadjuvant) local and systemic therapies, have provided a dramatic reduction in local recurrence rate and improved survival outcomes for patients with locally advanced rectal cancer (LARC) [2,3]. The most commonly used multidisciplinary approach for LARC patients is an intravenous or oral 5-fluorouracil (5FU)-based neoadjuvant chemoradiotherapy (nCRT) followed by a standardized surgical technique (total mesorectal excision) and postoperative adjuvant chemotherapy, which is the standard of care in Western countries [3,4,5]. This could lead to reduced local recurrence rate and improved disease-free survival, with approximately 20% of patients achieving a pathologic complete response (pCR: ypT0M0) at the time of surgery, whereas a considerable proportion of patients exhibit resistance to nCRT, thereby resulting in only minimal to no regression or disease progression, even during nCRT [6,7]. Such heterogeneous responses to nCRT among patients with LARC can finally impact long-term oncological outcomes [6,7,8,9]. It is also important to balance the risk of local and metastatic recurrence, avoiding over-treatment, preserving organ function and patients’ quality of life [3]. Accordingly, to determine the optimal treatment plan, there is a critical need of predictive biomarkers that can discriminate between non-responsive patients with LARC from those of responsive. Identifying potential non-responders and responders before neoadjuvant treatment may help clinicians to consider more personalized multidisciplinary strategies that include intensive preoperative treatment, such as total neoadjuvant therapy (TNT), upfront surgery to avoid unnecessary treatment-related toxicities, as well as non-operative “watch and wait” management [2,3].

Tumor tissue-based molecular predictors of response to nCRT in patients with LARC have been extensively studied using pre-treatment biopsy specimens. Particularly, many researchers reported gene expression signatures as predictive biomarkers for nCRT response based on high-throughput technologies, such as microarrays. However, in 2011, Brettingham-Moore et al. evaluated several gene signatures reported in earlier studies [10,11,12] (published between 2005 and 2008), and revealed that these published signatures had a limited accuracy in independent samples of LARC [13]. They thus concluded that gene expression-based signatures based on microarray analyses could not reliably predict nCRT response [13]. More recently, several promising classifiers based on gene expression have been reported [14,15,16,17,18,19,20,21,22]. These signatures were typically identified and developed in discovery or training cohorts and their predictive performance was confirmed in internal or external validation cohorts. It is important to note that despite the high prediction accuracy of those reported signatures, they had very few overlap in the identified genes among studies and also lacked prospective validation in clinical trials. Accordingly, none of the gene signatures are currently available for predicting nCRT response in patients with LARC in clinical practice.

The aim of this study was to build transcriptional multi-gene assays for predicting nCRT response in LARC, based on comprehensive screening in multiple datasets of pre-treatment biopsies. We also aimed at validating the predictive performance of various signatures, including ours and several previously-reported multi-gene and single-gene predictors reported since 2011. In order to test those signatures, we used an in silico meta-analysis approach based on six microarray datasets, which have been publicly available since 2016, of pre-therapeutic biopsies from patients with LARC that were independent of those used in developmental studies.

## 2. Materials and Methods

### 2.1. Microarray-Based Gene Expression Data Analysis

All expression datasets used in this study are publicly accessible in the Gene Expression Omnibus (GEO) database (http://www.ncbi.nlm.nih.gov/geo, accessed on 12 June 2021). We used the preprocessed values obtained from each microarray dataset. If a gene is represented by multiple probes, only the probe with the highest mean expression was used.

To calculate each gene signature, a representative score was determined as the sum of expression values for genes that are upregulated in non-responders minus the sum of expression values for genes that are downregulated in non-responders.

### 2.2. Selection of Microarray Datasets for Comprehensive Screening (the Discovery Cohorts)

For comprehensive screening of differentially expressed genes between non-responders and responders to nCRT, we obtained three microarray datasets of pre-therapeutic biopsies from LARC, including GSE35452, GSE45404 and GSE53781. They were each originally used to develop and validate multi-gene signatures predictive of nCRT response in three different studies, reported between 2014 and 2015.

### 2.3. Selection of Microarray Datasets for Validation (the Validation Cohorts)

We searched the GEO database in May 2021 with the search terms, (rectal cancer or rectal adenocarcinoma or rectal carcinoma) and (chemoradiotherapy or radiochemotherapy or chemoradiation or biopsy), filtered by series type (Expression Profiling by Array). Thirty-four GEO series identified by the initial GEO search were screened on the basis of eligibility criteria that required each dataset to be based on genome-wide expression data for pre-treatment biopsies from LARC patients who underwent nCRT and to include response information for more than 30 patients. We only included datasets that were published in 2012 or later. The resulting six independent datasets, including GSE46862, GSE68204, GSE94104, GSE119409, GSE133057, and GSE150082, were used as the validation cohorts.

### 2.4. Statistical Analysis

Unpaired *t*-test was used to determine differences between two groups. The discriminatory ability of different gene signatures was assessed in each dataset by performing the receiver operating characteristic (ROC) analysis based on continuous values for each signature, and areas under the ROC curve (AUCs) and their 95% confidence intervals (95% CI) were calculated as a measure of overall prediction accuracy. The pooled AUC was calculated using the fixed-effects model. Statistical heterogeneity between studies was assessed using I^2^ statistic. Statistical analyses were performed using GraphPad Prism v6.04 (Graphpad Software, San Diego, CA, USA), SPSS Statistics version 26 (IBM Corporation, Armonk, NY, USA), or EZR (Saitama Medical Center, Jichi Medical University, Saitama, Japan), a graphical user interface for R 2.6.1 (The R Foundation for Statistical Computing, Vienna, Austria). All statistical tests were two-sided, and *p* values less than 0.05 were considered statistically significant.

## 3. Results

### 3.1. Identification of Four Common Genes Differentially Expressed between Non-Responders and Responders in the Discovery Datasets

**Figure 1:**
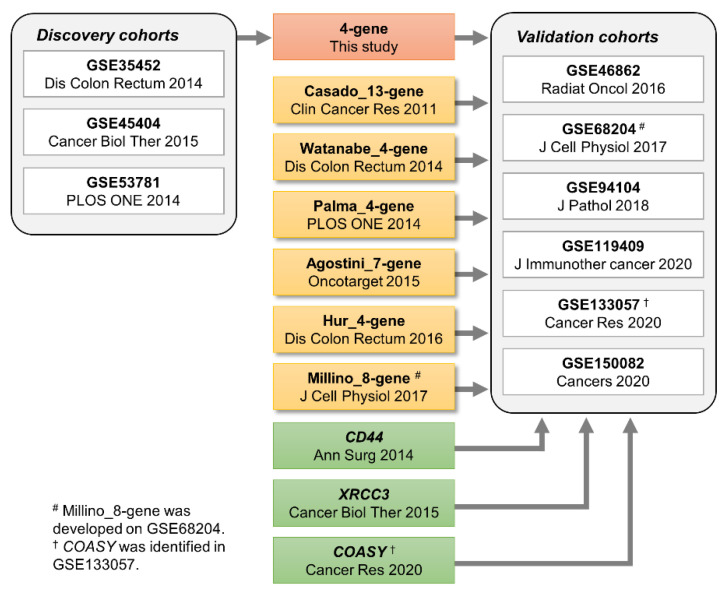
Overall study design. Three microarray datasets, published between 2014 and 2015, were used as the discovery cohorts to screen genes for building the 4-gene signature. The systematic search of the GEO database identified six microarray datasets as the validation cohorts to test the predictive performance of our signature and previously-published multi-gene or single-gene signatures.

The overall workflow of this study is illustrated in Figure 1. In 2011, Brettingham-Moore et al. critically reported that previously-published gene signatures had limited accuracy for predicting nCRT response [13]. Subsequently, in 2014, through 2015, multi-gene predictors for nCRT were reported, and microarray datasets of pre-treatment biopsies obtained from LARC patients, including GSE35452 (*n* = 46), GSE45404 (*n* = 42), and GSE53781 (*n* = 26) were deposited in the GEO database [14,15,18]. The present study utilized these three datasets as the discovery cohorts, consisting of a total of 61 non-responders and 53 responders, analyzed on Affymetrix or CodeLink platforms (Table 1). The detailed characteristics of the datasets are also demonstrated in Table S1.

**Figure 2:**
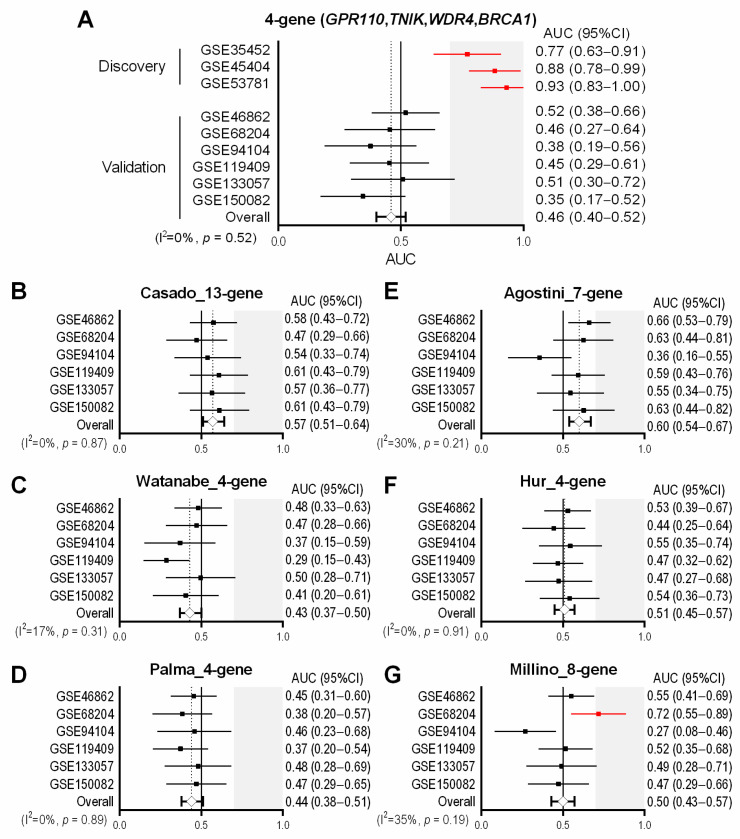
Assessment of the predictive performance of multi-gene signatures in the validation cohorts. (**A**–**G**) Forest plots of the area under the receiver operating characteristics curve (AUC) values and 95% confidence intervals (95% CI) for tested signatures, including the 4-gene (**A**), the Casado_13-gene (**B**), the Watanabe_4-gene (**C**), the Palma_4-gene (**D**), the Agostini_7-gene (**E**), the Hur_4-gene (**F**), and the Millino_8-gene (**G**). Datasets that were used to for the development of signatures (red squares and lines) were excluded from the meta-analyses.

With the use of the three discovery datasets, we first conducted comprehensive screening of genes that were differentially expressed between pre-therapeutic biopsies obtained from non-responders and responders. As demonstrated in Figure S1A,B four common genes, including G-protein-coupled receptor 110 *(GPR110*), TRAF2 and NCK interacting kinase *(TNIK*), WD repeat domain 4 *(WDR4*) and BRCA1 DNA repair associated *(BRCA1*), in those three different datasets were identified. In tumor biopsies from non-responders, *GPR110* and *TNIK* were significantly upregulated, while *WDR4* and *BRCA1* were significantly downregulated, as compared to those of responders (*p* < 0.05). We then generated an expression signature on the basis of those four genes calculated as ((*GPR110* + *TNIK*)—(*WDR4* + *BRCA1*)), showing higher levels of the 4-gene signature in biopsies from non-responders than those of responders with AUCs ranging from 0.77 to 0.93 in each of the discovery cohort (Figure 2A and Figure S1C).

### 3.2. Assessment of the Predictive Performance of the 4-Gene Signature in the Validation Cohorts

We then sought to determine the reproducibility of the predictive performance of our 4-gene signature in multiple independent datasets. For this purpose, the systematic search of the GEO database was conducted, and the search terms and the eligibility criteria were described in Materials and Methods, and in Figure S2. We identified six datasets of pre-therapeutic biopsies from LARC patients who underwent nCRT followed by surgical resection, including GSE46862 (*n* = 69), GSE68204 (*n* = 38), GSE94104 (*n* = 40), GSE119409 (*n* = 56), GSE133057 (*n* = 33), and GSE150082 (*n* = 39), each analyzed on different microarray platforms, including Affymetrix, Illumina, and Agilent (Figure 1 and Figure S2, Table 1 and Table S1). These six GEO datasets were used as the validation cohorts that contained a total of 176 non-responders and 99 responders [19,22,23,24,25,26]. However, the levels of the 4-gene signature did not significantly differ between non-responders and responders in any of the six cohorts analyzed (*p* > 0.05, Figure S3), showing no prediction accuracy to differentiate non-responders from responders with AUCs around 0.50 in each cohort (Figure S3 and Figure 2A). The pooled AUC was 0.46 (95% CI 0.40–0.52) in the meta-analysis for the six validation cohorts (Figure 2A). Although we further analyzed each of the four genes, *GPR110*, *TNIK*, *WDR4,* and *BRCA1*, at the single-gene level, it did not consistently differ between non-responders and responders in the validation cohorts (Figure S4).

### 3.3. Previously-Published Multi-Gene Signatures in the Validation Cohorts

Although our attempt to develop a gene signature predictive of nCRT response was unsuccessful, we next evaluated the predictive performance of six previously-reported multi-gene signatures that were identified from the literature published since 2011, as listed in Table 2 and illustrated in Figure 1. Signature scores were calculated as the sum of expression values for upregulated genes in non-responders minus the sum of expression values for downregulated genes in non-responders. For each of the six validation datasets, the levels of the six candidate signatures were examined in non-responders and responders (Figure S5A–F and Figure S6) and then AUC values and 95% CIs were plotted (Figure 2B–G). Since the Millino_8-gene was built based on GSE68204 [19], we confirmed reasonably higher levels of this signature score in non-responders compared to responders (Figure S5F) with an AUC of 0.72 (95% CI 0.55–0.89) in GSE68204 (Figure S6B and Figure 2G). Additionally, the Agostini_7-gene were significantly higher in non-responders in GSE46862 (*p* < 0.05, Figure S5D), showing an AUC of 0.66 (95% CI 0.53–0.79) (Figure S6A and Figure 2C). The Watanabe_4-gene in GSE119409 and the Millino_8-gene in GSE94104 showed statistically significant difference between non-responders and responders (*p* < 0.05, Figure S5B,F), but in the opposite direction (AUCs < 0.5, Figure S6C,D and Figure 2E,G). Collectively, except for the two analyses mentioned above (the Millino_8-gene in GSE68204 and the Agostini_7-gene in GSE46862), none of the six signatures showed significant higher levels in non-responders compared to responders in any of the validation cohorts (*p* > 0.05, Figure S5A–F), giving limited or no discriminative power with diverse AUCs ranging from 0.27 to 0.63 in each dataset (Figure 2B–G). Likewise, the meta-analyses demonstrated that those six signatures had limited predictive values with the pooled AUCs of less than 0.6, namely, 0.57 (95% CI 0.51–0.64) for the Casado_13-gene, 0.43 (95% CI 0.37–0.50) for the Watanabe_4-gene, 0.44 (95% CI 0.38–0.51) for the Palma_13-gene, 0.60 (95% CI 0.54–0.67) for the Agostini_7-gene, 0.51 (95% CI 0.45–0.57) for the Hur_4-gene, and 0.50 (95% CI 0.43–0.57) for the Millino_8-gene (Figure 2B–G).

### 3.4. Previously-Published Single-Gene Signatures in the Validation Cohorts

**Figure 3:**
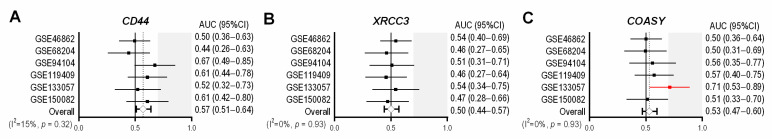
Assessment of the predictive performance of single-gene signatures in the validation cohorts. (**A**–**C**) Forest plots of the area under the receiver operating characteristics curve (AUC) values and 95% confidence intervals (95% CI) for tested genes, including *CD44* (**A**), *XRCC3* (**B**), and *COASY* (**C**). Datasets that were used to identify genes (red square and line) were excluded from the meta-analyses.

As demonstrated in Figure S7A–C, Figure S8A–F, and Figure 3A–C, we further tested three different single-gene signatures, including the expression of *CD44*, *XRCC3* and *COASY* (Table 2), using the six validation cohorts (Table 1). Since *COASY* was originally identified and reported using GSE133057 [22], we confirmed that the expression of *COASY* was significantly increased in non-responders compared to responders (*p* < 0.05, Figure S7C), showing an AUC of 0.71 (95% CI 0.53–0.89) in GSE133057 (Figure S8E and Figure 3C). However, none of the other analyses showed significant difference of each single-gene levels between non-responders and responders (*p* > 0.05, Figure S7A–C) and AUCs ranged from 0.44 to 0.67 (Figure S8A–F). As demonstrated in Figure 3A–C, the meta-analyses revealed that those three genes had limited predictive values with the pooled AUCs: 0.57 (95% CI 0.51–0.64) for *CD44*, 0.50 (95% CI 0.44–0.57) for *XRCC3*, and 0.53 (95% CI 0.47–0.60) for *COASY*.

## 4. Discussion

Here, we analyzed a total of nine microarray cohorts of pre-therapeutic biopsy specimens obtained from 389 patients with LARC who received nCRT followed by surgery. We initially built our own signature to be predictive of nCRT response based on comprehensive analyses in the three discovery datasets. Thereafter, using the six independent validation cohorts of pre-treatment biopsies, we evaluated the performance of the 4-gene signature we developed, as well as several transcriptional signatures that were previously-reported in the literature. Even though these signatures showed clear discrimination between non-responder and responders at least in their developmental cohorts, we found that none of the tested signature scores were consistently different between tumor specimens from non-responders and responders. This resulted in a weak or no discriminative ability of each signature in the pooled analyses. Overall, our findings do not support clinically-meaningful predictive values of those signatures on the basis of microarray data for responsiveness to nCRT in patients with LARC.

The present study identified a set of genes, including *GPR110*, *TNIK*, *WDR4,* and *BRCA1*, to be each significantly associated with nCRT response in the discovery cohorts of pre-treatment biopsies from LARC. It is worth noting that *BRCA1* plays a pivotal role in DNA repair and cell cycle regulation in response to DNA damage, potentially resulting from chemotherapy and radiotherapy. Indeed, several studies reported that *BRCA1* mRNA expression had predictive impact on responses to chemotherapy, as well as to chemoradiotherapy in many types of cancer, such as breast cancer, lung cancer, and esophageal cancer [27,28,29,30,31], suggesting *BRCA1* as an attractive candidate for predicting nCRT response in LARC. It has been reported that *GPR110* (*ADGRF1*) can induce cell cycle arrest and chemoresistance in breast cancer [32]. *TNIK* appeared to be an essential factor for WNT signaling and stemness in colorectal cancer [33] and might be responsible for chemoresistance in osteosarcoma [34]. However, the 4-gene signature, consisting of *GPR110*, *TNIK*, *WDR4,* and *BRCA1*, showed no association with nCRT response in any of the validation cohorts of LARC biopsies. Likewise, despite the promising correlations of the previously-reported signatures in the original publications [14,15,16,17,19,20], we found little evidence that they have sufficient clinical utility to guide decision-making in the current practice. This discrepancy could be due to small sized patient cohorts, technical differences between laboratories, disparities between various microarray probes and platforms, and insufficient analytical validation. It is also important to note that tumor biopsy-based transcriptomic profiles are likely prone to sampling bias due to intra-tumor heterogeneity. Moreover, there are differences and controversies in nCRT regimens, including chemotherapeutic drugs and radiation doses [2,3]. Optimal timing of surgical resection after nCRT also remains debatable, as longer intervals from nCRT to surgery appeared to lead to a greater potential of achieving a pCR in a meta-analysis [35], but delaying surgery was significantly associated with increased morbidity in a randomized trial [36]. Histological tumor regression grade is assessed using various grading systems with subjective categorization to reflect therapeutic response, leading to interobserver variability [37,38]. Such considerable variabilities among previously-published studies and datasets, including nCRT regimens, intervals from CRT to surgery, grading systems for tumor regression, and definitions of non-responder might also be a potential bias. We suggest that future gene signature studies of LARC using microarray or other expression platforms would be required to address those limitations.

In 2011, Brettingham-Moore et al. demonstrated that the microarray-based gene signatures, which were reported between 2005 and 2008, did not retain their predictive power in an independent cohort. Hence, they suggested that alternative approaches for predictive studies in LARC should be considered [13]. Although the present study again demonstrated that the gene expression signatures derived from microarray technologies were not capable of discriminating non-responders from responders, most recent studies have extensively searched for promising candidates to predict nCRT response in LARC using more robust expression assays and platforms. For instance, multi-gene expression assays developed on the NanoString nCounter system may provide more accurate prediction to nCRT response in LARC [39,40]. DNA-based molecular markers, including mutations in *KRAS* or *TP53* and microsatellite instability (MSI), may also be associated with responses to neoadjuvant treatment in LARC [41,42,43]. Other promising tissue-based predictive biomarkers, including the expression of microRNAs [38], differentially methylated CpGs [44], immune profiles [45,46], multi-protein expression assays by immunohistochemistry [47], are necessary to be validated on large, retrospective, and prospective cohorts of pre-treatment LARC biopsies, although the translation of biomarkers into clinical practice remains challenging. In addition, integrative approaches that include not only tissue-based biomarkers but also liquid-based molecular assays and imaging modalities may contribute to more sensitive stratification of patients with LARC receiving nCRT [2,38].

## 5. Conclusions

In this study, we developed a novel gene expression-based classifier for predicting nCRT response based on microarray cohorts of pre-treatment biopsies from patients with LARC. Although our signature and previously published signatures were tested in multiple independent datasets for validation purposes, none of them were capable of classifying patients with LARC into responders and non-responders to nCRT. We suggest that current gene expression-based signatures using microarray platforms are not robust enough to predict nCRT response or guide clinical decision-making in patients with LARC.

## Figures and Tables

**Table 1:** GEO datasets of pre-treatment biopsies from patients with LARC who underwent nCRT.

Purpose	GEO Accession	Platform	Total	Non- Responder	Responder	References
Discovery	GSE35452	Affymetrix Human Genome U133 Plus 2.0 Array	46	22	24	[14]
	GSE45404	Affymetrix Human Genome U133 Plus 2.0 Array	42	23	19	[18]
	GSE53781	CodeLink Human Whole Genome Array	26	16	10	[15]
Validation	GSE46862	Affymetrix Human Gene 1.0 ST Array	69	41	28	[23]
	GSE68204	Agilent-014850 Whole Human Genome Microarray 4x44K G4112F	38	22	16	[19]
	GSE94104	Illumina HumanHT-12 WG-DASL V4.0 R2 expression beadchip	40	29	11	[24]
	GSE119409	Affymetrix Human Genome U133 Plus 2.0 Array	56	41	15	[25]
	GSE133057	Illumina human-6 v2.0 expression beadchip	33	20	13	[22]
	GSE150082	Agilent-026652 Whole Human Genome Microarray 4x44K v2	39	23	16	[26]

**Table 2:** Multi-gene and single-gene expression signatures tested in meta-analyses.

Signature	Genes Upregulated in Non-Responders vs. Responders	Genes Downregulated in Non-Responders vs. Responders	References
4-gene	*GPR110* (*ADGRF1*), *TNIK*	*WDR4*, *BRCA1*	This study
Casado_13-gene	*BAK1*, *MLH1*, *TYMS*, *CKB*, *GPX2*, *HIG2* (*HILPDA*), *PH4* (*P4HTM*)	*ALDH1A1*, *CDKN1A*, *FOS*, *RELB*, *STAT3*, *TFF3*	[17]
Watanabe_4-gene	*FRMD3*, *SAMD5*, *TMC7*	*LRRIQ3* (*LRRC44*)	[14]
Palma_4-gene	-	*GNG4*, *MYC*, *POLA1*, *RRM1*	[15]
Agostini_7-gene	*AKR1C3*	*CXCL11*, *CXCL10*, *IDO1*, *CXCL9*, *MMP12*, *HLA-DRA*	[16]
Hur_4-gene	*TP53*	*MKI67*, *CDKN1A*, *CD133* (*PROM1*)	[20]
Millino_8-gene	*ITGA2*, *NRG1*, *KLF7*	*TMEM188* (*CNEP1R1*), *TRAM1*, *BCL2L13*, *MYO1B*, *GTSE1*	[19]
*CD44*	*CD44*	-	[21]
*XRCC3*	-	*XRCC3*	[18]
*COASY*	*COASY*	-	[22]

## Data Availability

The datasets used in this study are openly accessible through the Gene Expression Omnibus (GEO) database (http://www.ncbi.nlm.nih.gov/geo, accessed on 12 June 2021).

## Supplementary Materials

The following are available online at https://www.mdpi.com/article/10.3390/cancers13184642/s1, Figure S1: Identification of 4 common genes that were significantly differentially expressed between biopsy specimens from non-responders and responders in three discovery cohorts. Figure S2: Dataset selection flowchart for the validation cohorts. Figure S3: The performance of the 4-gene signature in six validation datasets. Figure S4: Differential expression analyses of four single genes in the 4-gene signature between non-responders and responders in nine cohorts. Figure S5: The levels of six multi-gene signatures in non-responders and responders in six validation cohorts. Figure S6: The performance of six multi-gene signatures in six validation cohorts. Figure S7: The levels of six multi-gene signatures in non-responders and responders in six validation cohorts. Figure S8: The performance of three single-gene signatures in six validation cohorts. Table S1: Characteristics of GEO datasets.
