# Supplementary material for: Validation of Gene Expression-Based Predictive Biomarkers for Response to Neoadjuvant Chemoradiotherapy in Locally Advanced Rectal Cancer
Source: Cancers (Basel). 2021 Sep 16;13(18):4642. doi: 10.3390/cancers13184642 (PMC8467397; doi:10.3390/cancers13184642)
Supplement: Supplementary file 1 [file cancers-13-04642-s001.zip › cancers-1324452-supplementary.pdf]

Supplementary Figure S1

A

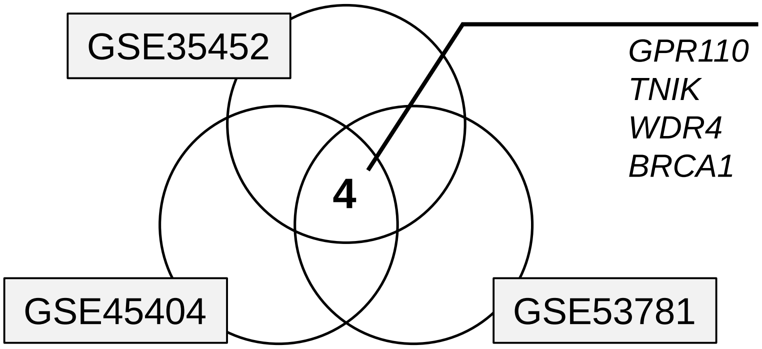

B

Gene Symbol	GSE35452				GSE45404				GSE53781			
	Non-Responder (n=22)	Responder (n=24)	Log2 fold-change	*P*	Non-Responder (n=23)	Responder (n=19)	Log2 fold-change	*P*	Non-Responder (n=35)	Responder (n=11)	Log2 fold-change	*P*
GPR110	0.14	-1.06	1.21	0.048	3.25	2.81	0.44	0.011	0.45	-1.02	1.47	0.009
TNIK	0.21	-0.45	0.66	0.005	4.83	4.13	0.70	0.024	0.44	-0.29	0.73	0.019
WDR4	-0.22	0.32	-0.54	0.033	5.39	5.87	-0.48	0.031	1.39	2.41	-1.02	0.020
BRCA1	-0.08	0.19	-0.27	0.037	5.31	5.67	-0.37	0.006	1.33	2.55	-1.22	0.024

C

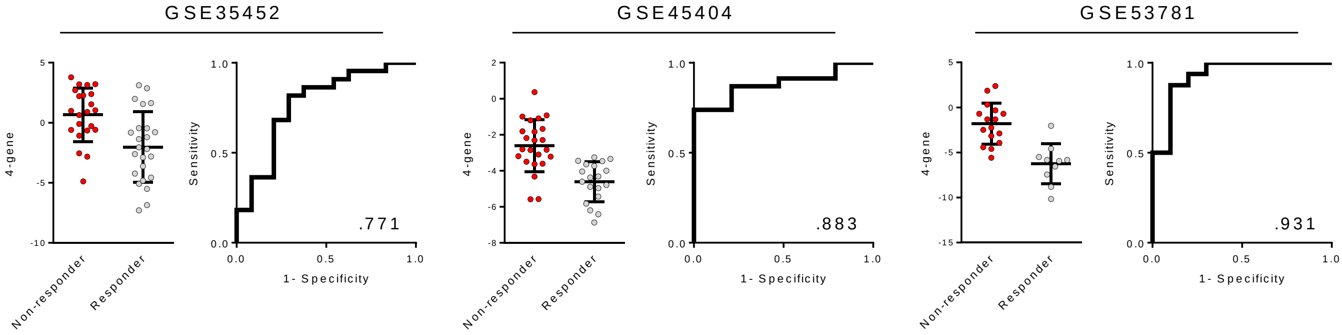

Supplementary Figure S1. Identification of 4 common genes that were significantly differentially expressed between biopsy specimens from non-responders and responders in three discovery cohorts, including GSE35452, GSE45404 and GSE53781. (A,B) The expression of GPR110 and TNIK was increased, but WDR4 and BRCA1 expression was decreased in non-responders compared to responders. (C) The levels of the 4-gene signature based on GPR110, TNIK, WDR4 and BRCA1 were higher in non-responders with AUCs ranging between 0.77 and 0.93 in each of the discovery cohort.

Supplementary Figure S2

GEO database

- Search: rectal cancer OR rectal adenocarcinoma OR rectal carcinoma) AND (chemoradiotherapy OR radiochemotherapy OR chemoradiation OR biopsy (May 2021)
- Filter: Expression profiling by array

34 datasets

Eligibility

- Published in 2012 or later
- Pretreatment biopsy samples from patients with LARC who underwent nCRT followed by surgery
- A minimum of 30 samples with available information on responses to nCRT
- Comprehensive mRNA expression data
- Datasets used in the discovery cohorts were excluded
- Microdissected samples were excluded

6 datasets as the validation cohorts

Supplementary Figure S2. Dataset selection flowchart for the validation cohorts. 34 datasets identified by GEO search were evaluated. Six datasets were selected to be included in the discovery cohorts.

Supplementary Figure S3

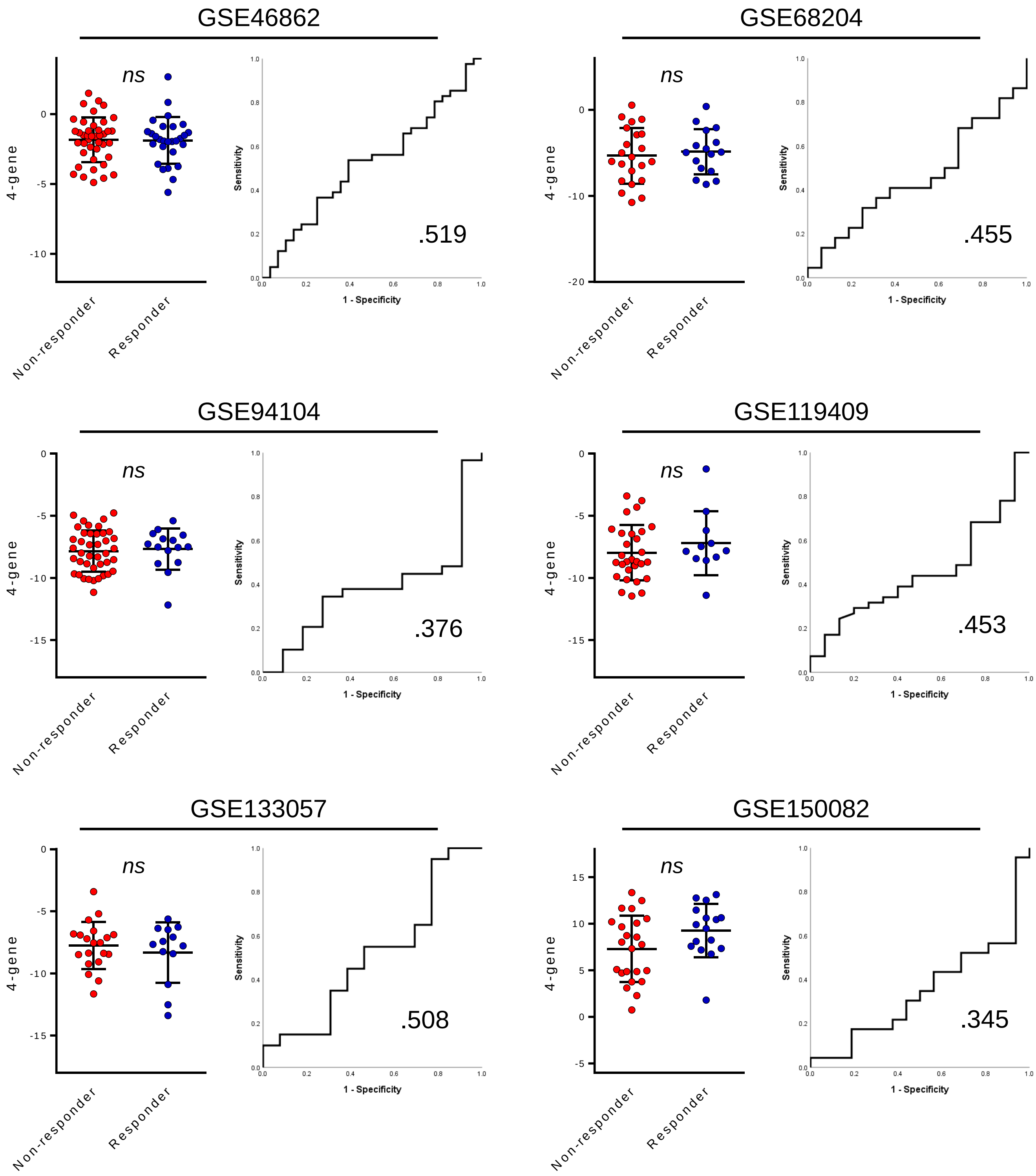

Supplementary Figure S3. The performance of the 4-gene signature in six validation datasets.

Supplementary Figure S4

	Discovery			Validation					
Dataset:	GSE35452	GSE45404	GSE53781	GSE46862	GSE68204	GSE94104	GSE119409	GSE133057	GSE150082
GPR110	0.048	0.011	0.009	0.737	0.342	0.433	NA	0.411	0.715
TNIK	0.005	0.024	0.019	0.294	0.258	0.812	0.756	0.569	0.159
WDR4	0.033	0.031	0.020	0.572	0.266	0.792	0.786	0.730	0.020
BRCA1	0.037	0.006	0.024	0.601	0.263	0.172	0.483	0.809	0.079

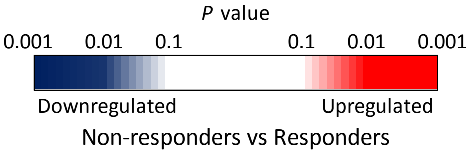

Supplementary Figure S4. Differential expression analyses of four single genes in the 4-gene signature between non-responders and responders in nine cohorts. *P* values are indicated for each analysis, and the red or blue colors represent upregulation or downregulation of genes in non-responders.

Supplementary Figure S5

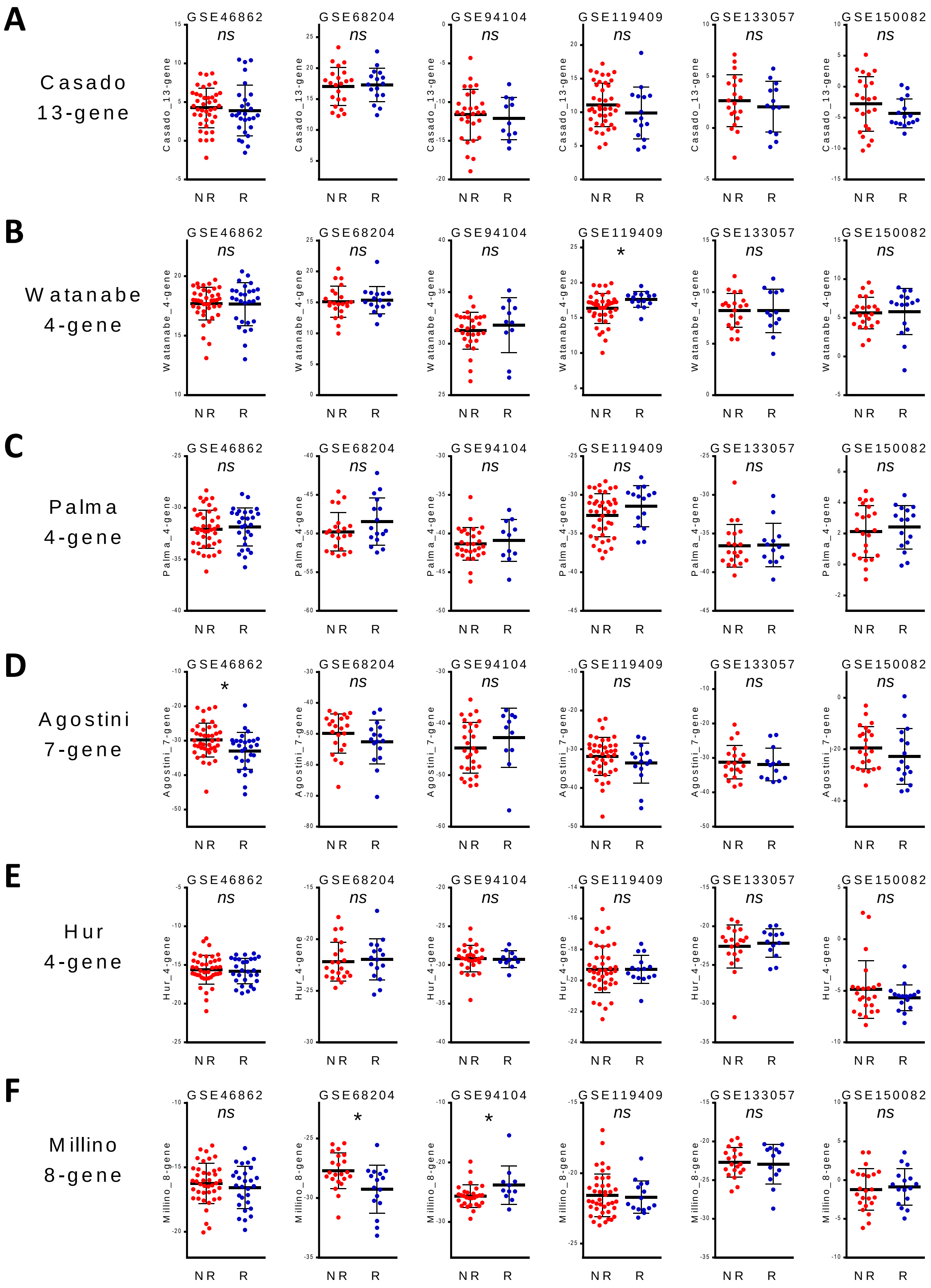

Supplementary Figure S5. The levels of six multi-gene signatures in non-responders (NR) and responders (R) in six validation cohorts.

Supplementary Figure S6

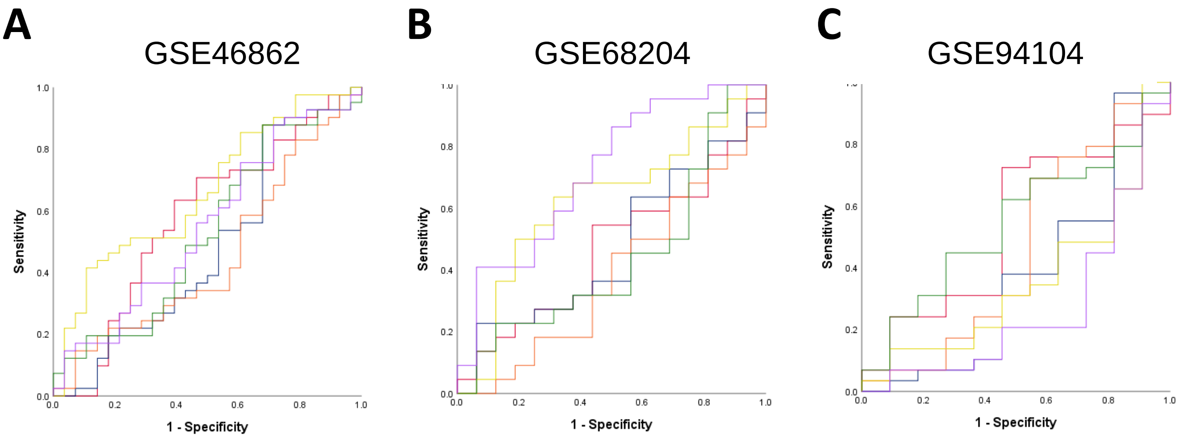

	AUC (A)	AUC (B)	AUC (C)
Casado_13-gene	.575	.472	.539
Watanabe_4-gene	.481	.472	.370
Palma_4-gene	.454	.384	.458
Agostini_7-gene	.662	.625	.357
Hur_4-gene	.530	.443	.545
Millino_8-gene	.551	.719	.270

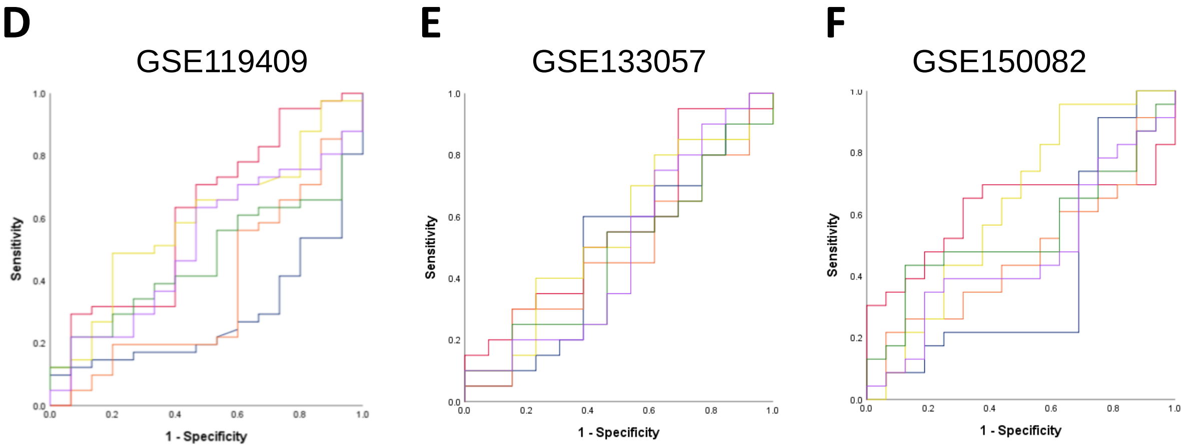

	AUC (D)	AUC (E)	AUC (F)
Casado_13-gene	.608	.565	.611
Watanabe_4-gene	.287	.496	.405
Palma_4-gene	.372	.481	.470
Agostini_7-gene	.594	.546	.628
Hur_4-gene	.470	.473	.541
Millino_8-gene	.517	.492	.473

Supplementary Figure S6. The performance of the multi-gene signatures in six validation cohorts.

Supplementary Figure S7

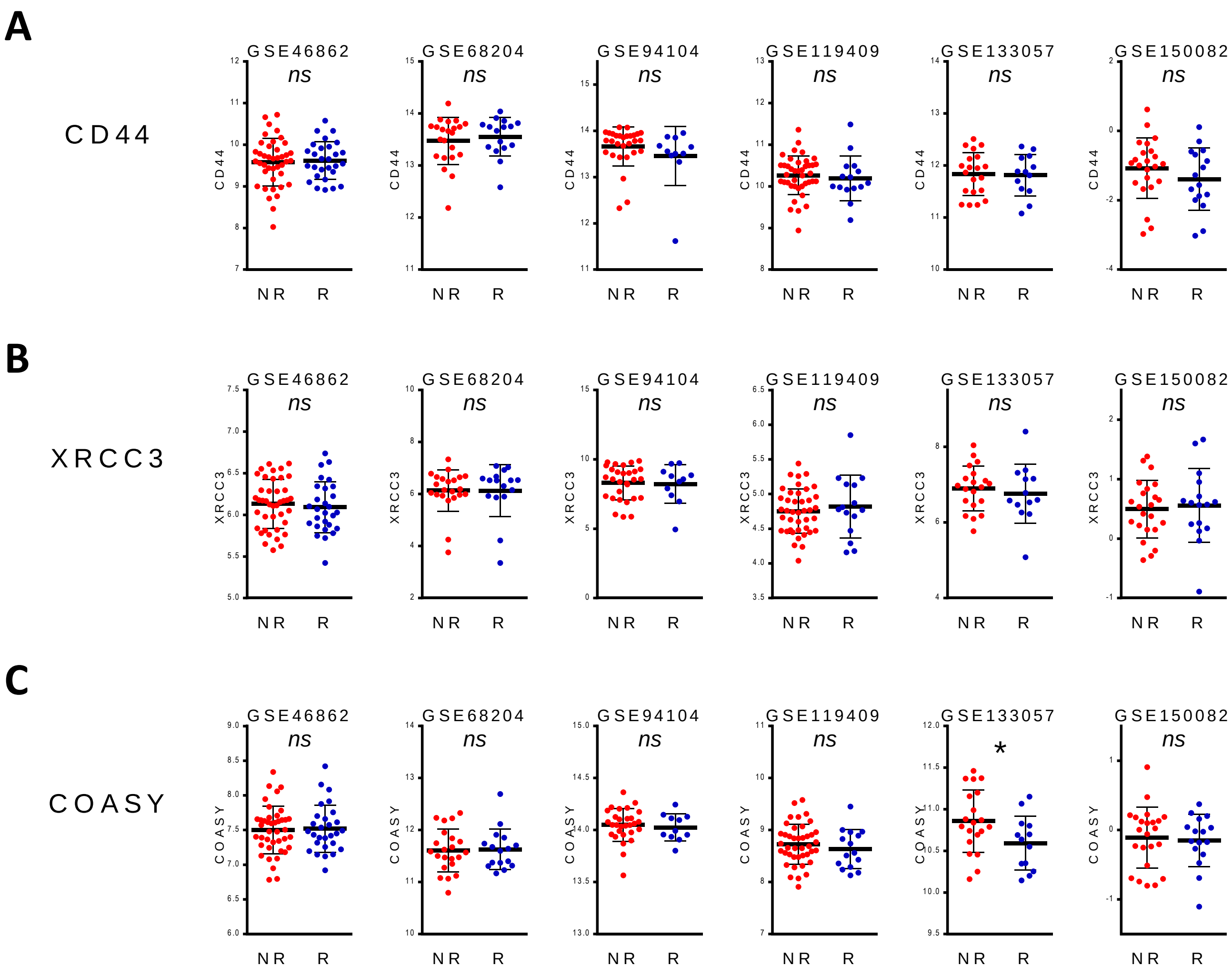

Supplementary Figure S7. The levels of six multi-gene signatures in non-responders (NR) and responders (R) in six validation cohorts.

Supplementary Figure S8

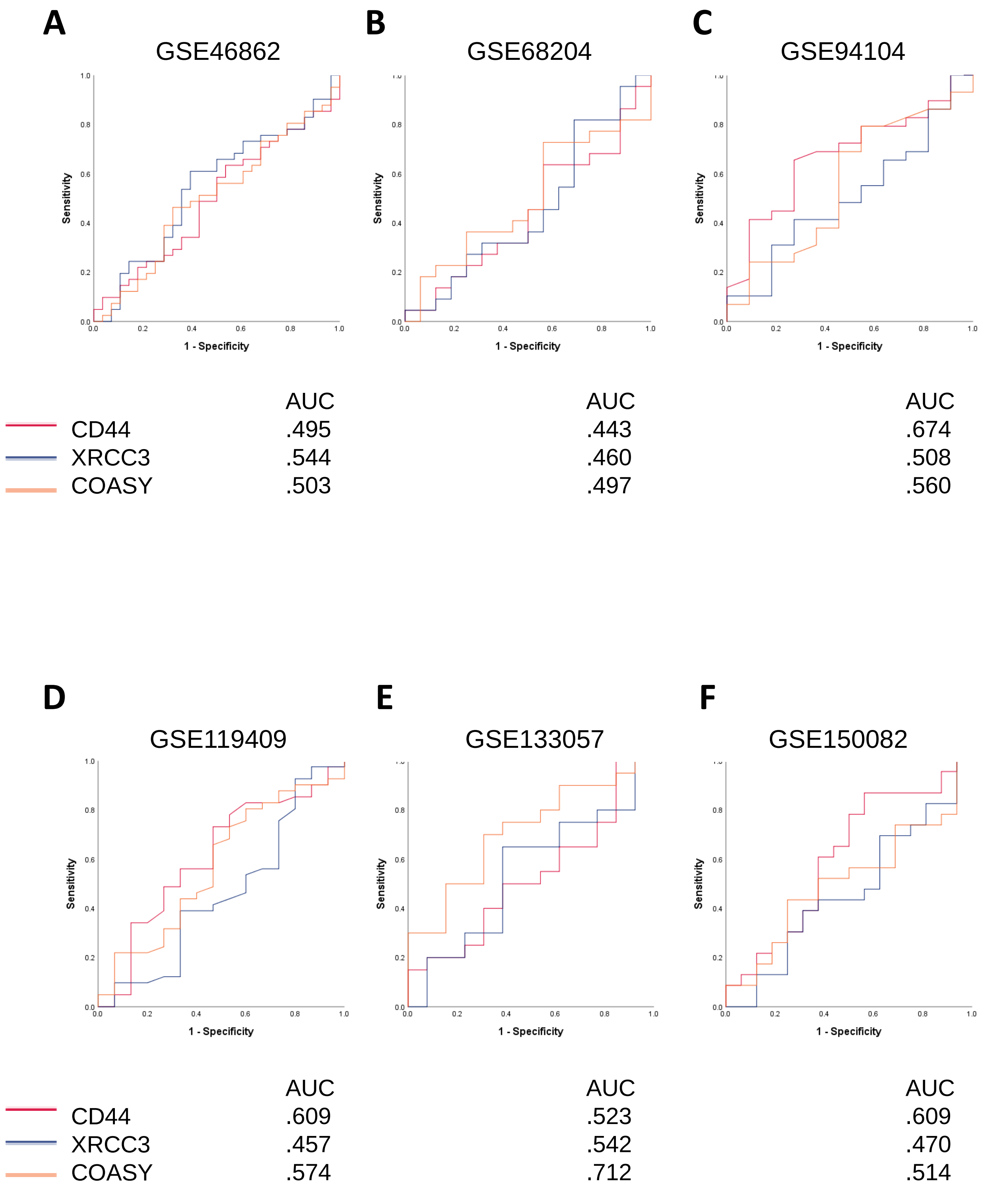

Supplementary Figure S8. The performance of three single-gene signatures in six validation cohorts.

Supplementary Table S1. Characteristics of GEO datasets.

GEO accession	sample type	nCRT regimen	Age (Mean ± SD)	Sex	Non-responders according to sample characteristics reported in each GEO dataset	Responders according to sample characteristics reported in each GEO dataset	Tumor regression grading (TRG) system
**GSE35452**	pre-treatment biopsy/frozen	UFT/LV with RT 50.4Gy	N/A	N/A	"Non-responder" =grade0-1	"Responder" =grade2-3	The Japanese Society for Cancer of the Colon and Rectum (grade 0-3)
**GSE45404**	pre-treatment biopsy/frozen	5FU iv with RT >50Gy	59.9 ± 11.1	24 male 18 female	"Non-responder" =TRG 3-5	"Responder" =TRG 1-2	Mandard (TRG 1-5)
**GSE53781**	pre-treatment biopsy/frozen	Cape ± L-OHP with RT 50.4Gy	60.6 ± 11.7	7 male 18 female	"Non-responder" =TRG 3-5	"Responder" =TRG 1-2	Mandard (TRG 1-5)
**GSE46862**	pre-treatment biopsy/frozen	N/A	55.7 ± 10.8	49 male 20 female	grade 0-2	grade 3-4	Dworak (grade 0-4)
**GSE68204**	pre-treatment biopsy/frozen	5FU iv or Cape ± L-OHP with RT >45Gy	64.5 ± 8.4	25 male 13 female	"Non-responder" =TRG 3-5	"Responder" =TRG 1-2	Mandard (TRG 1-5)
**GSE94104**	pre-treatment biopsy/FFPE	5FU iv or Cape with RT 45Gy	N/A	N/A	TRG 1-2	TRG 3	Four-tier tumor regression grade system (TRG 0-3)
**GSE119409**	pre-treatment biopsy/frozen	Cape with RT 50.6Gy	56.8 ± 11.1	N/A	"Resistant"	"Sensitive"	Four-tier tumor regression grade system (TRG 0-3)
**GSE133057**	pre-treatment biopsy/frozen	5FU iv with RT 50.4Gy	56.8 ± 10.4	23 male 10 female	AJCC score 2-3	AJCC score 0-1	The AJCC criteria (AJCC 0-3)
**GSE150082**	pre-treatment biopsy/frozen	Cape with RT 50.4Gy or Cape + L-OHP followed by Cape with RT 50.4Gy	58.8 ± 11.0	29 male 10 female	"Poor response" =TRG 2-3	"Good response" =TRG 0-1	American Joint Committee on Cancer and College of American Pathologists regression grade
